# Assessment of Antibody Stability in a Novel Protein-Free Serum Model

**DOI:** 10.3390/pharmaceutics13060774

**Published:** 2021-05-22

**Authors:** Joachim Schuster, Vinay Kamuju, Roman Mathaes

**Affiliations:** Lonza Pharma and Biotech, Drug Product Services, 4057 Basel, Switzerland; joachim.schuster@lonza.com (J.S.); vinay.kamuju@lonza.com (V.K.)

**Keywords:** protein stability, monoclonal antibodies, in vitro model, developability, physiological conditions

## Abstract

Therapeutic proteins can degrade upon administration as they are subjected to a variety of stresses in human body compartments. In vivo degradation may cause undesirable pharmacokinetic/pharmacodynamic profiles. Pre-clinical in vitro models have gained scientific interest as they enable one to evaluate the in vivo stability of monoclonal antibodies (mAbs) and ultimately can improve patient safety. We used a novel approach by stripping serum of endogenous proteins, which interfere with analytical test methods. This enabled the direct analysis of the target protein without laborious sample work-up procedures. The developed model retained the osmolality, conductivity, temperature, and pH of serum. We compared the impact of human, bovine, and artificial serum to accelerated stability conditions in histidine buffer. Target mAbs were assessed in regard to visible and sub-visible particles, as well as protein aggregation and fragmentation. Both mAbs degraded to a higher extent under physiological conditions compared to accelerated stability conditions. No relevant stability differences between the tested mAbs were observed. Our results reinforced the importance of monitoring protein stability in biological fluids or fluids emulating these conditions closely. Models enabling analysis in fluids directly allow high throughput testing in early pre-clinical stages and help in selecting molecules with increased in vivo stability.

## 1. Introduction

Therapeutic proteins such as monoclonal antibodies (mAbs) can degrade after administration to patients [1,2]. Once injected or infused, mAbs are subjected to sudden changes as human biological fluids/tissues differ fundamentally from drug product formulations. mAbs are subjected to a macromolecule-rich matrix and undergo changes in conditions such as temperature, pH, ionic composition, and osmolality [1]. Moreover, the stabilizing excipients administered along with the mAb degrade/diffuse away. Such changes may impact protein stability and, in turn, can have ramifications on their efficacy and/or safety profile. In recent years, in vitro models appeared as a promising tool to evaluate the stability of potential lead candidates under physiological conditions. These invitro models allow one to predict the impact on critical quality attributes upon administration into a human body compartment [3].

Different in vitro models and analytical approaches have been developed to evaluate the in vivo stability of therapeutic proteins [4,5,6,7]. However, simulating physiological conditions and detecting the protein of interest in biological fluids is accompanied by challenges [8,9]. Purification techniques and/or fluorescence labeling have been successfully used to detect the target protein [10,11]. However, both methods can be laborious and potentially impact the intrinsic protein properties and stability. Instead, biological fluids can be substituted with buffer systems that enable analysis in fluids directly yet deviate markedly from the conditions found in vivo (e.g., phosphate-buffered saline) [6,12,13]. Alternatively, stripping the neat biological fluid of macromolecules can lead to higher resemblance of the physiological conditions encountered.

In this study, we used centrifugal ultrafiltration to strip human and bovine serum of macromolecules >3 kDa. We simulated intravenous (IV) administration and assessed the impact on protein stability by comparing human serum filtrate (HSF), bovine serum filtrate (BSF), artificial serum (AS), and histidine buffer under accelerated stability conditions. The stability of two mAbs was monitored over 14 days by light obscuration (LO), high performance-size exclusion chromatography (HP-SEC), and capillary electrophoresis–sodium dodecyl sulfate (CE-SDS).

## 2. Materials and Methods

### 2.1. Materials

Proprietary mAb1 (pI 9.1) and mAb2 (pI 8.1) were obtained from Lonza Biologics, Slough, UK and formulated at 10 mg/mL, 20 mM histidine buffer, pH 6.0. Buffers for analytical methods and in vitro models were prepared using alpha-D glucose, calcium chloride, histidine, L-histidine monohydrochloride monohydrate, potassium chloride, magnesium chloride hexahydrate, sodium bicarbonate, sodium chloride, sodium phosphate monobasic monohydrate, and sodium phosphate dibasic. All buffer components were purchased from Sigma-Aldrich (St. Louis, MO, USA). Fetal bovine serum (BS) and human serum (HS) were obtained from Gibco (ThermoFischer Scientific, Waltham, MA, USA) and Gemini Bio-Products (West Sacramento, CA, USA). Pierce™ Protein Concentrator PES, 3 kDa molecular weight (MW) cut-off, 5–20 mL was purchased from ThermoFischer Scientific. All samples were prepared and incubated in 10 mL sterile Nalgene cryoware (PETG media bottles, Sigma-Aldrich).

### 2.2. Preparation of Fluids

Four fluids were prepared; namely, 20 mM histidine buffer (pH 6.0), AS, BSF, and, HSF. AS and serum filtrates were prepared at pH 7.4. Sera were stripped of macromolecules by centrifugal ultrafiltration (3 h, 2000 rpm) with a 3 kDa protein concentrators. Protein concentrators were primed with 2.5 mL of MilliQ H_2_O prior usage. AS contains electrolytes (127 mM Na^+^, 111 mM Cl^−^, 25 mM HCO_3_^−^, 5 mM K^+^, 3.75 mM Ca^2+^) and 5 mM glucose to match the values provided by the vendor of human serum and the literature [14]. CaCl_2_ was added to AS after adjusting the pH to 7.4 with CO_2_ to avoid Ca_3_(PO_4_)_2_ and CaCO_3_ precipitation [15]. All fluids were filtered using a 0.22 µm PES filter (Merck Millipore, Darmstadt, Germany) before use.

### 2.3. Physiological In Vitro Model

Intravenous administration was simulated by spiking mAb1 and mAb2 in 4 mL of the aforementioned fluids to obtain a final concentration of 0.5 mg/mL, as used in a previous in vitro model [16]. Blank controls were prepared by spiking each fluid with a 20 mM histidine buffer without a mAb. The 20 mM histidine buffer samples (pH 6.0) were incubated in a chamber under accelerated stability conditions, i.e., 40 °C and 75% relative humidity. AS, BSF, and, HSF samples were maintained at pH 7.4 by incubating at 37 °C and 6.5% CO_2_. Samples were analyzed after 30 min (T0d), 7 days (T7d), and, 14 days (T14d) of incubation. The incubation period was selected based on previous studies [17]. All samples and controls were characterized in regard to visible particles, pH, sub-visible particles (SbVP), protein aggregation, and, fragmentation. All samples and controls were prepared in triplicates. The values were compared to reference material (RM) of mAb1 and mAb2, stored in 20 mM histidine buffer (pH 6.0) at 5 °C.

### 2.4. pH, Conductivity and Osmolality

pH and conductivity were measured using SevenExcellence pH and conductivity meter (Mettler Toledo, Columbus, OH, USA). The osmolality of each fluid was determined using a freezing point OsmoPRO micro-osmometer (Advanced instruments, Norwood, MA, USA).

### 2.5. Visible Particles

Each sample and control were inspected for visible particles according to the method described in the European Pharmacopoeia (2.9.20).

### 2.6. Light Obscuration

SbVPs were analyzed using HIAC 9703+ equipped with a HRLD-400 detector (Beckman Coulter, Brea, CA, USA) and a 1 mL Hamilton syringe, at a flow rate of 10 mL/min. SbVPs ≥2, ≥5, ≥10, and, ≥25 µm in size were characterized using the LO method described in the European Pharmacopoeia (2.9.19); however, with a modified volume of 0.2 mL. Each sample was measured four times and the data from last three measurements were averaged and reported. Data was acquired using the PharmSpec software (Beckman Coulter, Brea, CA, USA). The instrument was calibrated using 5 µm COUNT-CAL Count Precision Size Standard beads (Thermo Fisher Scientific, Waltham, MA, USA).

### 2.7. Protein Content

The protein concentration of mAbs and fluids was determined using variable path spectroscopy. SoloVPE, C Technologies (Bridgewater Township, NJ, USA) mounted onto an Agilent Cary 60 UV–Vis spectrometer (Santa Clara, CA, USA). Samples were measured at 280 nm in fibrettes. Data was acquired by Cary WinUV software 5.0.0.1008 (Agilent Technologies, Santa Clara, CA, USA). The mAb concentration in BSF and HSF was determined by subtracting the absorbance of each blank control from the serum samples spiked with a mAb.

Protein content was also estimated using the Pierce™ Coomassie Plus (Bradford) Assay Kit (ThermoFischer Scientific, Waltham, MA, USA). For measurements, Coomassie Plus Reagent was mixed with samples and incubated for 10 min at room temperature. The absorbance of the samples was measured at 595 nm using Lico 690 spectrophotometer (Hach, Loveland, CO, USA). Protein concentration was determined using an albumin calibration curve according to the manufacturer.

### 2.8. High Performance Size Exclusion Chromatography

Protein monomer, high-molecular weight species (HMWS), and, low-molecular weight species (LMWS) were monitored by HP-SEC. Samples were analyzed using a TSKgel GS3000SWXL column (Tosoh Biosciences, Griesheim, Germany) and a Waters Alliance e2695 HPLC system equipped with a UV–Vis detector. The instrument was flushed with mobile phase consisting of 0.2 M sodium phosphate buffer (pH 7.0) at a flow rate of 0.5 mL/min. Samples (15 µL) were detected at 214 nm. The obtained chromatograms were processed with Empower3 software (Waters, Milford, MA, USA).

### 2.9. Capillary Electrophoresis–Sodium Dodecyl Sulfate

Samples were analyzed by CE-SDS using LabChip GXII (Caliper Life Sciences, Hopkinton, MA, USA). Samples were prepared using a Protein Clear^TM^ HR Reagent kit (Caliper Life Sciences, Hopkinton, MA, USA). The reducing sample buffer was prepared by mixing 1 M dichlorodiphenyltrichloroethane with Protein Clear HR sample buffer, while the non-reducing buffer consisted of only Protein Clear HR sample buffer. Samples were mixed with reducing or non-reducing buffer and denatured at 70 °C for 10 min. Samples were centrifuged at 2000 rpm for 2 min and diluted with MilliQ H_2_O prior to analysis. The peaks between 16 to 250 kDa were processed using the Empower3 software (Waters).

## 3. Results

### 3.1. Development of an In Vitro Protein Free Serum Model

**Figure 1:**
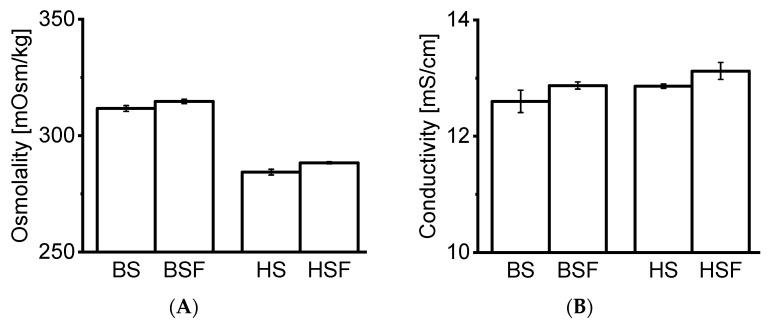
Preparation of protein-free serum. (**A**) Osmolality (**B**) conductivity for native sera and filtrates. *n* = 3. All bars expressed as mean ± standard deviation.

BSF and HSF were prepared from neat BS and HS using centrifugal ultrafiltration. BSF and HSF were colorless, although BSF appeared slightly cloudy. Neat sera and associated filtrates were compared in regard to osmolality and conductivity. Osmolality and conductivity were negligibly higher in filtrates (Figure 1A,B). The pH of the filtrates remained in the physiological range of 7.4 ± 0.2 over 14 days. The osmolality and conductivity of AS was 264 ± 0.8 mOsm/kg and 14.11 ± 0.08 mS/cm.

The filtrates were measured by spectroscopy and Bradford assay. Bradford assay indicated the complete absence of proteins in the filtrates as no absorbance was measured. However, spectroscopy showed absorbance, suggesting the presence of a low proteinaceous material remaining in the filtrate.

**Figure 2:**
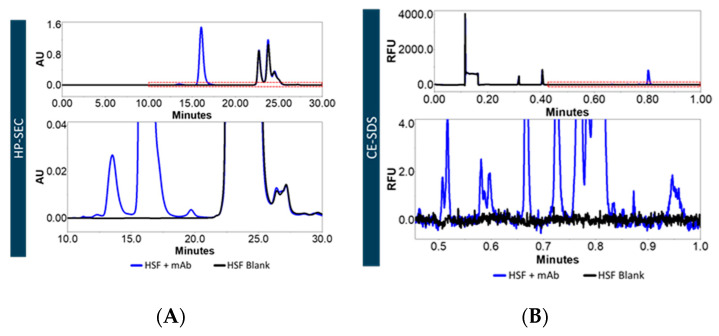
Analytics of protein-free serum. Filtrates present no interference to analytical measurements. HSF is shown as representative filtrate. (**A**) Chromatogram of HP-SEC of a mAb spiked into HSF (HSF+mAb) and HSF blank (without a mAb). (**B**) Electropherogram for non-reduced CE-SDS for mAb spiked into HSF (HSF+mAB) and HSF blank (without a mAb). For CE-SDS only peaks between 16 and 250 kDa (corresponding to 0.4 to 1.0 min) can be measured reliably and are presented in the magnified view (bottom panel). The blue line represents HSF+mAb, the black line denotes HSF blank. AU, absorbance units. RFU, relative fluorescence units. mAb1 spiked into HSF was chosen as representative sample.

As a next step, we evaluated whether the filtrate matrix interfered with the detection of a spiked mAb by HP-SEC and CE-SDS. Figure 2A shows the chromatogram for HSF spiked with mAb and HSF blank. The peaks from the HSF blank started at approx. 22 min, well beyond the peaks of the mAbs and its fragments, thereby allowing for the distinct detection of mAb fragments. The same observation was made for BSF (data not shown). CE-SDS measurements (Figure 2B) also showed no interference from the HSF blank. Consequently, the BSF and HSF were applicable to monitor the stability of mAbs.

### 3.2. Visible and Sub-Visible Particle Analysis

**Figure 3:**
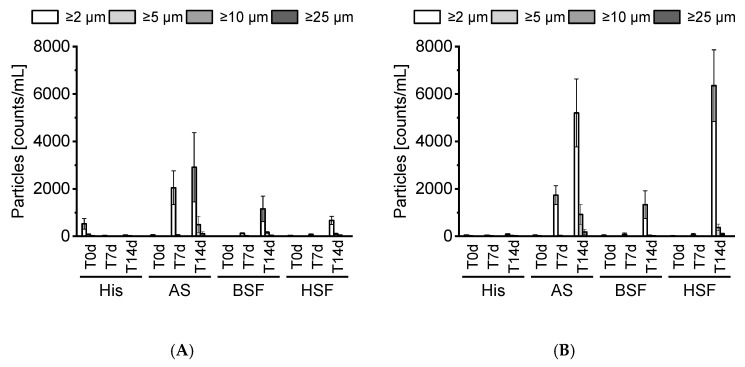
SbVP analysis. SbVP count for mAb1 (**A**) and mAb2 (**B**). Each sample was prepared in triplicates and measured once at each time point. *n* = 3. Cumulative SbVPs counts ≥2, ≥5, ≥10, and, ≥25 µm in size were characterized. All bars expressed as mean ± standard deviation. His, histidine buffer; AS, artificial serum; BSF, bovine serum filtrate; HSF, human serum filtrate.

SbVPs of both mAbs were monitored over 14 days (Figure 3A,B). LO data revealed minor particle formation for either mAb under accelerated stability conditions. SbVP counts of both mAbs consistently increased in AS, BSF, and, HSF over time. Particles were predominantly < 5 µm. mAb2 formed a higher number of SbVPs in HSF than mAb1. The spiked mAb concentration was consistent among all samples (Figure S1). All fluid blanks showed negligible particles and were stable over 14 days. No visible particle formation was observed.

### 3.3. HP-SEC Analysis

**Figure 4:**
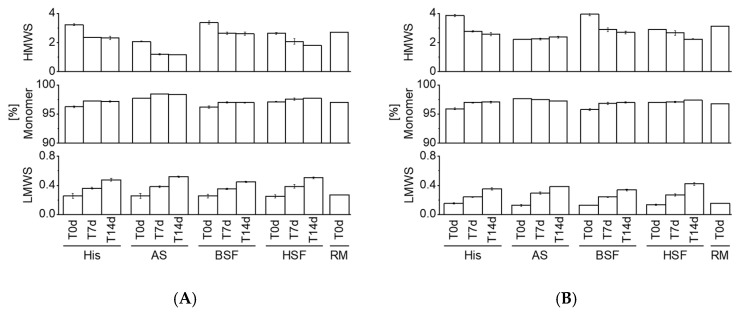
HP-SEC analysis. Measurements of mAb1 (**A**) and mAb2 (**B**) in four fluids over 14 days. All bars except for RM at T0d are expressed as mean with standard deviation. *n* = 3. His, histidine buffer; AS, artificial serum; BSF, bovine serum filtrate; HSF, human serum filtrate; RM, reference material. HMWS, monomer, and, LMWS are expressed as % area under the curve.

High-molecular weight species (HMWS), monomer, and, low-molecular weight species (LMWS) were characterized using HP-SEC (Figure 4A,B). HMWS of both mAbs decreased over time, with the exception of mAb2 in AS, wherein it remained unchanged. In contrast, LMWS of both mAbs consistently increased in all fluids. No substantial differences were observed among different fluids and mAbs. The variability of the samples was low (small error bars). Figure S2 shows a representative chromatogram over all time points.

### 3.4. Protein Fragmentation Analysis by CE-SDS

**Figure 5:**
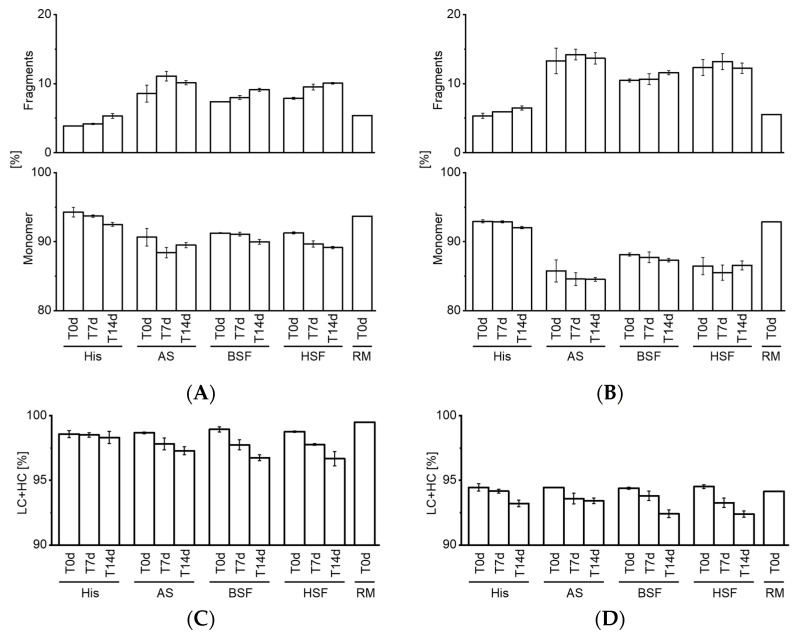
CE-SDS analysis. Non-reduced CE-SDS measurements for mAb1 (**A**) and mAb2 (**B**). Reduced CE-SDS measurements for mAb1 (**C**) and mAb2 (**D**). All bars except for RM at T0d are expressed as mean with standard deviation. *n* = 3. His, histidine buffer; AS, artificial serum; BSF, bovine serum filtrate; HSF, human serum filtrate; RM, reference material; LC + HC (%), light chain + heavy chain (%).

CE-SDS was used to analyze the purity of mAbs over time. The results for non-reduced CE-SDS are depicted in Figure 5A,B. Protein fragmentation was displayed in all four fluids. Protein fragments were markedly higher in AS, BSF, and, HSF compared to accelerated stability conditions and RM at T0d. Fragmentation appeared to be more prominent for mAb2 compared to mAb1. However, no striking increase in fragments was observed over 14 days for mAb2. Figure 5C,D (reduced CE-SDS) showed a consistent decrease over time. No substantial stability differences between the tested mAbs were observed by reduced CE-SDS in the fluids.

## 4. Discussion

Serum/plasma is the liquid component of blood, containing thousands of proteins and low-molecular weight molecules such as peptides, electrolytes, and, amino acids [18]. The protein concentration of 55 to 80 mg/mL in serum [19] prevents one from monitoring the stability of protein of interest (e.g., spiked mAb). Analytical approaches to overcome this challenge include the enrichment of the protein of interest (e.g., immunoaffinity purification) [20] or labeling (e.g., fluorescence dye) [5,11]. Alternatively, the biological fluid can be substituted with an appropriate surrogate buffer [6,12].

We present an alternative approach by stripping serum of endogenous proteins. We demonstrated that mAbs can be analyzed directly in serum filtrates without additional sample work-up procedures. The Bradford assay did not allow one to determine the protein content, presumably because molecules <3 kDa such as small peptides do not bind to the dye [21]. Precise protein concentration measurement via spectroscopy was not possible as the extinction coefficient of the remaining macromolecules in the filtrate was unknown. HP-SEC data indicated the presence of molecules of approx. ≤ 1–3 kDa in serum blanks, most likely peptides and aromatic amino acids. This is in agreement with our CE-SDS data, as no matrix interference from serum blanks was observed. Osmolality and conductivity values of the serum filtrates were negligibly higher than in neat serum, indicating that the electrolyte concentration remained similar. The composition of the serum filtrates was not further characterized as it was out of the scope of this study.

Kinderman et al. showed that protein candidates with poor colloidal stability under physiological conditions may aggregate or precipitate in vivo and thus, particles (visible and sub-visible) should be assessed during pre-clinical development [22]. During administration, a protein experiences sudden changes from the formulation conditions to those encountered in patients. This sudden transition can be simulated by pH jump assays in vitro [22]. In this study, the tested mAbs formed no visible particles. SbVPs were detected by LO under simulated physiological conditions. mAb2 appeared less stable compared to mAb1 and formed substantial SbVPs in HSF over time. The protein-free sera enabled the inspection of particles, which is typically not possible with biological fluids due to their poor stability in vitro [5,13,23]. Our prepared protein-free sera remained stable over 14 days and displayed a negligible particle count.

HP-SEC data showed a consistent increase in LMWS of both mAbs in all fluids over time. HMWS decreased consistently over time with the exception of mAb2 which remained within the same range in AS over 14 days. Previous studies reported an increase in HMWS under simulated physiological conditions [5,12]. Comparison to other studies is challenging due to differences in the analytical approach, protein concentration, fluids, sampling pull points, and, molecules. Our results suggest that certain protein aggregates may dissolve under physiological conditions. A variety of factors such as ionic composition, temperature, pH, etc. could contribute to the fate of protein aggregates in vivo [1]. Knowledge on in vivo protein aggregation is generally limited as it remains unknown if administered particles (e.g., formed over the shelf-life duration or during clinical preparation) dissolve in vivo or persist. Future studies could aim at exploring if the nature of protein particles formed during manufacturing and/or clinical preparation impacts the fate in vivo. A previous study by Filipe et al. reported dissimilar protein aggregation behavior in serum by comparing particles generated under different stress conditions [7]. Our CE-SDS results confirmed an increase in fragments in all fluids over time. Interestingly, comparing the RM to T0d values showed that protein fragments increased strikingly upon exposure to AS, BSF, and, HSF. This increase at T0d was not observed under accelerated stability conditions. Fragmentation was also more pronounced in AS, BSF, and, HSF compared to accelerated stability conditions.

Both mAbs degraded to a similar extent under physiological conditions, whereas they remained fairly stable under accelerated stability conditions. Particularly, SbVP formation and protein fragmentation were dissimilar under accelerated stability conditions and simulated physiological conditions. This reaffirms the importance of assessing the in vivo protein stability under a simulated physiological environment. Despite the differences in SbVP formation of mAbs in HSF, no relevant differences were observed between AS, BSF, and, HSF. Although all fluids remained stable, the cloudy appearance of BSF could hamper certain analyses (e.g., visible particle inspection) and appeared less suitable as a physiological in vitro model compared to AS and HSF. Artificial fluids such as AS may form SbVPs due to salt precipitation and therefore would be less applicable for particle analysis [12,15]. Fluids such as AS and the prepared filtrates, which are devoid of macromolecules, enable direct analysis in fluids; however, the matrix composition and osmolality of the prepared filtrates are more reflective of the physiological environment compared to AS. By stripping molecules based on molecular weight, theoretically, certain endogenous molecules such as peptides, nutrients (amino acids, lipids, carbohydrates), electrolytes, as well as small organic molecules and thiols remain in the filtrate [14]. Compared to neat serum, fluids devoid of cells and macromolecules such as enzymes may underestimate the impact on a protein’s stability.

Selecting a higher MW cut-off (e.g., 50 kDa) may retain certain serum proteases which can impact protein degradation. While this improves the physiological relevance of the model, the presence of enzymes could accelerate protein degradation. On the other hand, the presence of macromolecules can limit the stability of the fluid itself and cause analytical interference [5,13]. Thus, depending on the analytical methods used, future studies may select a higher MW cut-off to increase the physiological relevance while still allowing analysis in the fluids directly. Our developed protein-free serum remained stable over two weeks and may enable even longer incubation periods to detect protein liabilities among candidates. Moreover, avoiding sample work-up procedures such as purification or labeling is advantageous as they can be laborious and may alter the stability of a therapeutic protein. Overall, AS and protein-stripped serum appeared suitable to screen protein liabilities in vitro.

## 5. Conclusions

We have shown that centrifugal ultrafiltration enables one to strip serum of proteins while maintaining the physiological temperature, pH, electrolyte composition, and, osmolality. The developed in vitro model enabled one to monitor protein stability under physiological conditions without additional sample manipulation for the discrimination of the target mAbs. In this study, we focused on critical quality attributes such protein aggregation and fragmentation. Circumventing sample work-up procedures or additional dilution steps is particularly beneficial when assessing protein particles. Theoretically, protein-free sera maintain molecules smaller than the desired MW cut-off and thus resembles the physiological conditions closely. We showed that both mAbs degraded to a higher extent under physiological conditions as compared to accelerated stability conditions. This reinforced the importance to evaluate the in vivo stability under physiological conditions to screen potential protein liabilities. Future studies should correlate in vitro stability data to clinical samples from patients, which has been rarely attempted [6]. Notwithstanding, in vitro models are beneficial for pre-clinical studies to determine in vivo protein liabilities. Early knowledge of in vivo liabilities enables one to engineer superior molecule candidates and ultimately can lead to therapeutic proteins with increased safety and efficacy profiles. The study provides an essential step towards development of a reliable in vitro model.

## Data Availability

All data presented in this study are included in the submitted manuscript.

## Supplementary Materials

The following are available online at https://www.mdpi.com/article/10.3390/pharmaceutics13060774/s1, Figure S1: protein concentration of mAbs, Figure S2: HP-SEC chromatogram.
