# Supplementary material for: Assessment of Antibody Stability in a Novel Protein-Free Serum Model
Source: Pharmaceutics. 2021 May 22;13(6):774. doi: 10.3390/pharmaceutics13060774 (PMC8224624; doi:10.3390/pharmaceutics13060774)
Supplement: Supplementary file 1 [file pharmaceutics-13-00774-s001.zip › pharmaceutics-1203311-supplementary.pdf]

pharmaceutics

Supplementary Materials: Assessment of Antibody Stability in a Novel Protein-Free Serum Model

Joachim Schuster, Vinay Kamuju and Roman Mathaes

A

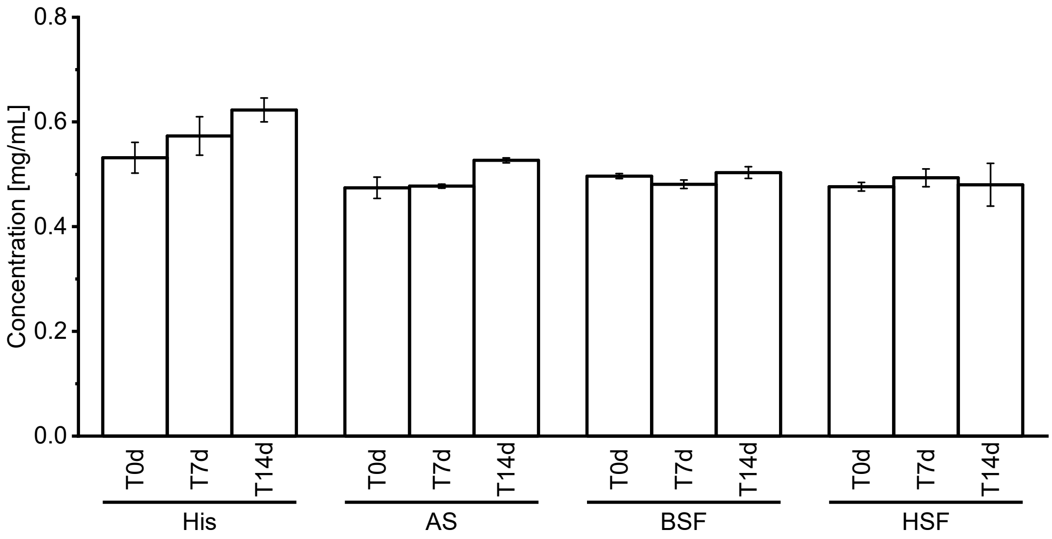

B

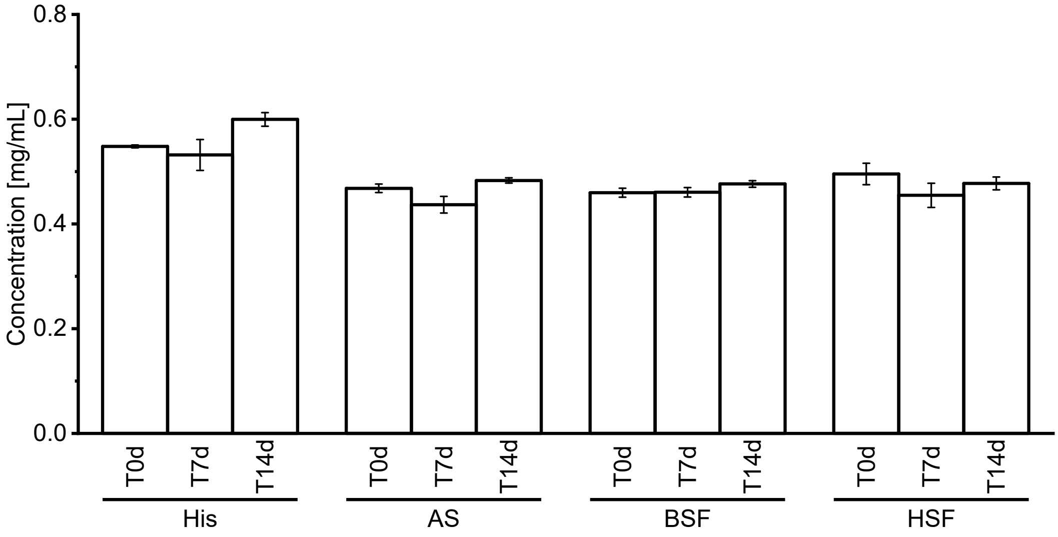

Figure S1. Protein concentration of mAbs: Protein concentration of mAb1 (**A**) and mAb2 (**B**) spiked into different fluids. For BSF and HSF, the protein concentration was determined by subtracting the contribution from the blank control samples. n=3. His, histidine buffer; AS, artificial serum; BSF, bovine serum filtrate; HSF, human serum filtrate.

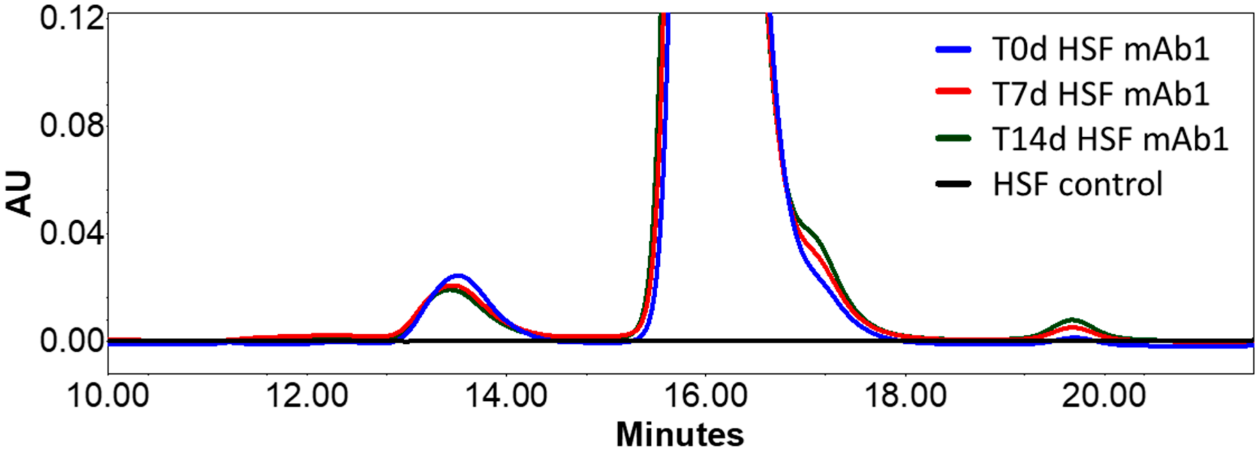

Figure S2. HP-SEC chromatogram. Overlay of T0d, T7d, T14d for mAb1 spiked into HSF and HSF blank at T0d. AU, absorbance units at 210 nm**.** HSF, human serum filtrate.
